# Antiestrogen-binding site ligands induce autophagy in myeloma cells that proceeds through alteration of cholesterol metabolism

**DOI:** 10.18632/oncotarget.1066

**Published:** 2013-06-11

**Authors:** Brigitte Sola, Marc Poirot, Philippe de Medina, Sophie Bustany, Véronique Marsaud, Sandrine Silvente-Poirot, Jack-Michel Renoir

**Affiliations:** ^1^ Normandie Univ, UNICAEN EA4652, Caen, France; ^2^ INSERM UMR1037, Centre de Recherche en Cancérologie de Toulouse, Toulouse, France; ^3^ Université de Toulouse III, Toulouse, France; ^4^ Institut Claudius Regaud, Toulouse, France; ^5^ Affichem, Toulouse, France; ^6^ Institut Curie, CNRS UMR3347, INSERM U1021, Orsay, France; ^7^ Institut Gustave Roussy, INSERM U749, Villejuif, France

**Keywords:** multiple myeloma, tamoxifen, autophagy, apoptosis, cholesterol metabolism

## Abstract

Multiple myeloma (MM) is a malignancy characterized by the accumulation of clonal plasma cells in the bone marrow. Despite extensive efforts to design drugs targeting tumoral cells and their microenvironment, MM remains an incurable disease for which new therapeutic strategies are needed. We demonstrated here that antiestrogens (AEs) belonging to selective estrogen receptor modulators family induce a caspase-dependent apoptosis and trigger a protective autophagy. Autophagy was recognized by monodansylcadaverin staining, detection of autophagosomes by electronic microscopy, and detection of the cleaved form of the microtubule-associated protein light chain 3. Moreover, autophagy was inhibited by drugs such as bafilomycin A1 and 3-methyladenosine. Autophagy was mediated by the binding of AEs to a class of receptors called the antiestrogen binding site (AEBS) different from the classical estrogen nuclear receptors. The binding of specific ligands to the AEBS was accompanied by alteration of cholesterol metabolism and in particular accumulation of sterols: zymostenol or desmosterol depending on the ligand. This was due to the inhibition of the cholesterol-5,6-epoxide hydrolase activity borne by the AEBS. We further showed that the phosphoinositide 3-kinase/AKT/mammalian target of rapamycin pathway mediated autophagy signaling. Moreover, AEBS ligands restored sensitivity to dexamethasone in resistant MM cells. Since we showed previously that AEs arrest MM tumor growth in xenografted mice, we propose that AEBS ligands may have a potent antimyeloma activity alone or in combination with drugs used in clinic.

## INTRODUCTION

Multiple myeloma (MM) is a B-cell malignancy characterized by the accumulation of clonal tumoral plasma cells in the bone marrow. The accumulation of malignant cells that synthesize immunoglobulins causes hyperproteinemia, renal dysfunction, bone lesions and immunodeficiency [1]. This disease still remains incurable despite novel therapeutic approaches targeting both myeloma cells and their bone marrow environment [2]. Selective estrogen receptor modulators (SERMs) and selective estrogen receptors disruptors (SERDs) or pure antiestrogens (AEs) may provide a potent strategy in myeloma therapy. Indeed, several groups, including our, have previously reported that SERMs and SERDs inhibited cell proliferation and/or induced apoptosis of MM cells [3-7]. Although myeloma cells express estrogen receptors (ER) belonging to both α and β isotypes [4, 5],it is not clear if AEs signal through canonical ERs. Nevertheless, *in vivo* experiments with xenografted mice bearing MM tumors clearly demonstrated that the 4-hydroxy-tamoxifen (OHT) as well as the pure AE, RU 58668 block tumor growth [8, 9]. This strongly supports that SERMs and SERDs may provide an alternative therapy for MM patients.

Tamoxifen (Tam) and its active metabolite, OHT are the prototypes of SERMs. They are high affinity ligands for nuclear ERs but also for other targets that can account for their biological activities [10]. Among them, is the microsomal antiestrogen binding site (AEBS) [11]. The AEBS results from hetero-dimerization of 3β-hydroxysteroid-Δ^8^-Δ^7^-isomerase and 3β-hydroxysteroid-Δ^7^-reductase both involved in the cholesterol biosynthesis pathway [11]. In addition, the AEBS carries out cholesterol-5,6-epoxide hydrolase (ChEH) activity [12]. ChEH catalyzes the transhydration of 5,6α-epoxy-cholesterol (5,6α-EC) and 5,6β-epoxy-cholesterol (5,6β-EC) into cholestane-3β,5α,6β-triol (CT) [13]. The AEBS/ChEH binds various structural classes of ligands: SERMs, several σ receptor ligands, polyunsaturated fatty acids and ring B oxysterols but neither estrogens nor the SERDs such as RU 58688 and fulvestrant [13].We have previously reported that AEBS-binding by Tam or OHT induces MCF7 breast cancer (BC) cell apoptosis and autophagy through the alteration of cholesterol metabolism [14]. Indeed, cholesterol precursors (5α-cholest-8-en-3β-ol, zymostenol and 5α-cholest-5,24-dien-3β-ol, desmosterol for tamoxifen and OHT treatments, respectively) accumulate in SERM-treated cells as the consequence of inhibition of cholesterogenic enzymes involved in ChEH/AEBS activity. We report here that: a) OHT induces apoptosis and autophagy in human multiple myeloma cell lines (HMCLs); b) OHT-treatment leads to the accumulation of free sterols in HMCLs due to the inhibition of the catalytic activity of the AEBS subunits and ChEH activity; c) AEBS ligands are responsible for cholesterol metabolism alteration in HMCLs and induction of autophagy. Taken in concert with *in vivo* activity of OHT [8], our data support a therapeutic potential of OHT and more generally AEBS ligands for antimyeloma therapy.

## RESULTS

### OHT triggers the intrinsic apoptotic pathway in HMCLs

**Figure 1:**
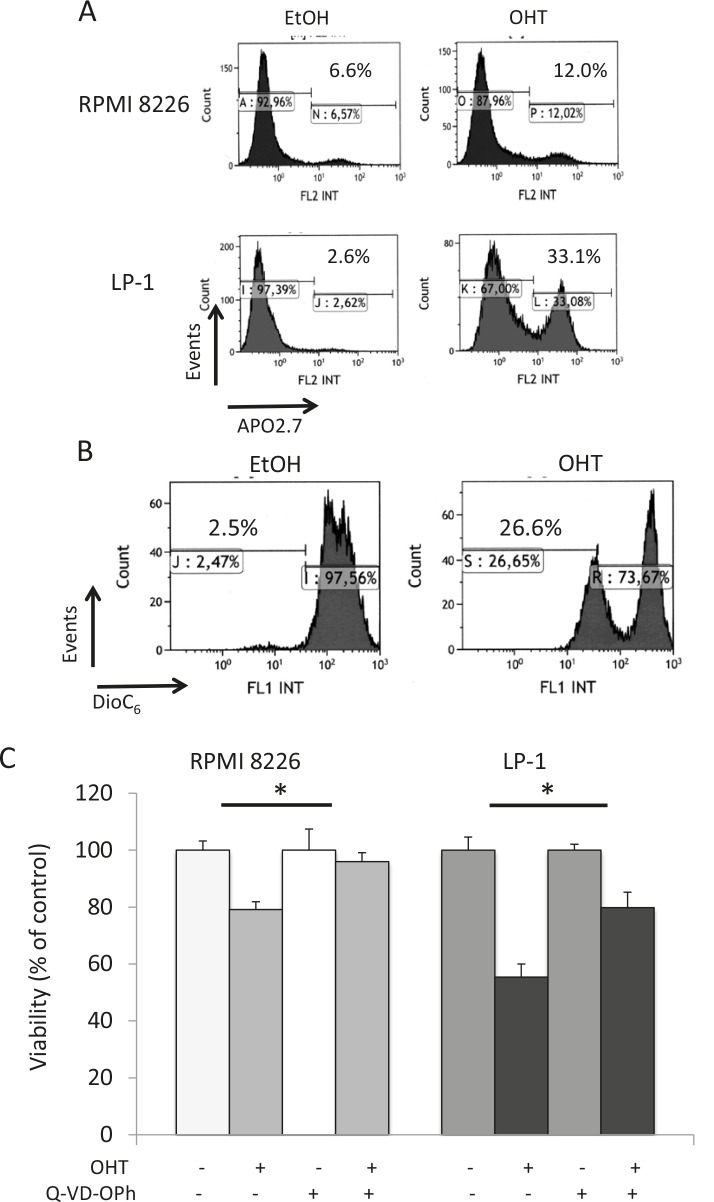
Caspase-dependent apoptosis is not the only form of active cell death occurring in OHT-treated HMCLs A) Exponentially growing RPMI 8226 and LP-1 cells were treated with EtOH for control or OHT (10 μM for 72 h) and apoptotic cells were analyzed after APO2.7 staining and cytometry sorting. The percentage of apoptotic cells (APO2.7-stained) is indicated on the graph. B) LP-1 cells were treated with vehicle or 10 μM OHT for 48 h and stained with DiOC_6_(3) before cytometry sorting. For cytometry analyses, an average of 2 × 10^5^ cells was sorted for each culture condition. C) RPMI 8226 and LP-1 cells were cultured with (or without) 10 μM Q-VD-OPh for 2 h, then treated with 10 μM OHT for 48 h. The number of viable cells was then determined with a MTT assay. Each experimental condition was repeated five times; the experiment was repeated twice. Plotted values are the percentage of viable cells referring to control experiments assigned to 100%. *, *p*<0.05.

We have previously reported that AEs belonging to both SERD and SERM classes display anti-proliferative and/or pro-apoptotic properties on MM cell lines and primary cells [5, 7]; these effects are dependent on AEs and cell lines (Supplementary Table 1). Roughly, OHT induces both G1 arrest and apoptosis in responsive HMCLs, while pure AEs such as RU 58668 and fulvestrant induce either G1 arrest or apoptosis, suggesting that each AE subtype does not affect identical pathways. We focused here on the biological effects of OHT in responsive HMCLs. As exemplified Figure 1A, APO2.7-positive RPMI 8226 and LP-1 cells were recognized after OHT treatment (10 μM for 72 h), indicating that cells underwent apoptosis. We confirmed the induction of apoptosis in OHT-treated cells by a double annexin V/propidium iodide staining and cytometry sorting (data not shown). When triggered, the OHT-induced apoptosis proceeded through the intrinsic mitochondrial pathway. This was associated with activation of upstream/downstream caspases [5],downregulation or cleavage of anti-apoptotic BCL2 family members (data not shown), loss of mitochondrial membrane potential (ΔΨm, Figure 1B), and, finally, cleavage of poly (ADP-ribose) polymerase [5]. However, when the caspase 3 inhibitor Q-VD-OPh was used in combination with OHT in HMCLs, we observed only a partial restoration of cell viability (Figure 1C). Our data suggest that another programmed cell death pathway, independent of caspases, was active in AE-treated HMCLs. Since autophagy was recognized as a regulator of both cell viability and death in MM [15], we looked for markers of autophagy in AE-treated cells.

### OHT induces autophagy in HMCLs

**Figure 2:**
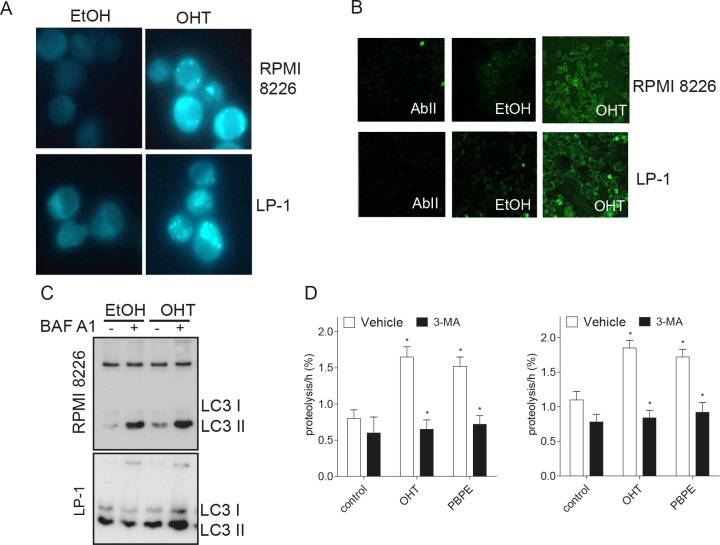
OHT-treatment induces ACD in MM cells A) Exponentially growing RPMI 8226 and LP-1 cells were treated with EtOH as vehicle or OHT at 10 μM for 24 h and stained with MDC. MDC-positive vesicles were observed with a confocal microscope (x400 magnification). B) Cells were treated with EtOH or OHT (10 μM) for 24 h and analyzed by transmission electron microscopy. C) RPMI 8226 and LP-1 cells were treated for 6 h with 10 μM OHT or vehicle, then subjected to immunocytochemistry with AbII alone as control or anti-LC3 Ab. Slides were analyzed by confocal microscopy (x180 magnification). D) RPMI 8226 and LP-1 cells were treated with 10 μM OHT (or EtOH) for 48 h followed by BAF A1 for 4 h (+) or not (-). Whole cell lysates were then analyzed by Western blotting with anti-LC3 Ab. D) Degradation of long-time proteins was determined in RPMI 8226 and LP-1 treated with EtOH, 10 μM OHT, 40 μM PBPE or 10 μM RU 58668 for 18 h in the presence or in the absence of 10 mM 3-MA. Experiments were repeated at three times in duplicate with comparable results. The data presented here are the means ± SEM of all experiments. *, *p*<0.001.

Macroautophagy (hereafter referred as autophagy) is characterized by the appearance of acidic vesicles that could be stained with monodansylcadaverin (MDC) and observed by fluorescence microscopy. As shown Figure 2A, OHT-treatment (10 μM for 24 h) caused the appearance of MDC-stained vesicles in HMCLs. We next analyzed the ultrastructural morphology of treated cells. In both cell lines, OHT-treatment (10 μM for 24 h) induced mitochondria alterations and condensation of chromatin confirming that the intrinsic apoptotic signaling pathway was triggered (Supplementary Figure 1). Autophagy is often accompanied by a massive cytoplasmic vacuolization that indicates increased autophagic flux [16]. Accordingly, OHT-treatment caused the formation of intracytoplasmic vesicles such as multilamellar bodies (MLBs), vacuoles surrounded by a double-membrane characteristic of autophagosomes and autolysosomes (Supplementary Figure 1). In sharp contrast, vehicle-treated cells lacked MLBs and autophagosomes (Supplementary Figure 1). The examination of cells at high magnification allowed us to notice that unilamellar vesicles (UVs) were present in OHT-treated HMCLs (Supplementary Figure 1, Table 1) suggesting an alteration of lipid metabolism. Microtubule-associated protein light chain 3 (LC3) is translocated to autophagosomes after lipidation and cleavage (LC3-I to LC3-II) at the onset of autophagy [17]. Indirect immunofluorescence was used for monitoring this phenomenon. As shown Figure 2B, OHT-treatment induced the translocation of LC3 to autophagosomes recognized by the appearance of green dots in both HMCLs. Moreover, OHT-treatment triggered the conversion of LC3-I into LC3-II, the latter being increased by a bafilomycin A1 (BAF A1) treatment as observed by immunoblotting (Figure 2C), evidencing that OHT induced an autophagic flux. We next showed that OHT stimulated the rate of long-lived protein degradation in both cell lines confirming the induction of autophagy (Figure 2D). Although a significant degree of basal autophagy was present in HMCLs (Figure 2C, Supplementary Figure 1), in agreement with a previous report [15], our data establish unambiguously that OHT induces an autophagic process.

### OHT and AEBS ligands compromise cholesterol metabolism in HMCLs

**Figure 3:**
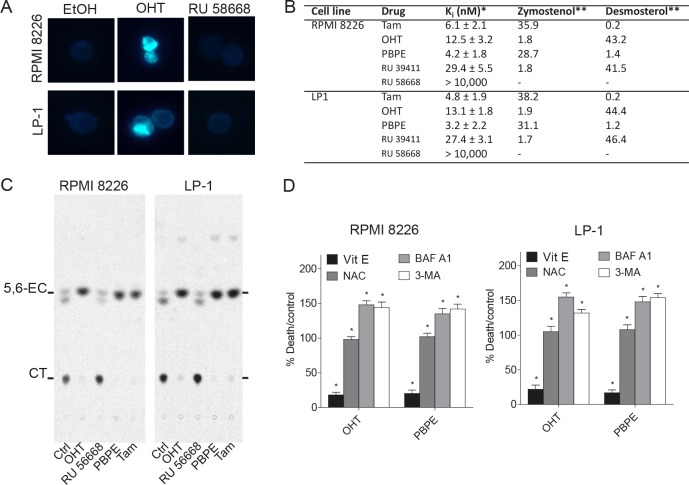
OHT and AEBS ligands induce the accumulation of cholesterol precursors in HMCLs A) RPMI 8226 and LP-1 cells were treated with EtOH or OHT (10 μM) for 48 h and cytospinned. Slides were stained with filipin and analyzed. Cells containing free sterols are colored in blue. Cells were visualized in the ultraviolet range using a x40 objective on a Zeiss LSM 510 microscope (Göettingen, Germany). B) The sterols accumulated in RPMI 8226 and LP-1 cells after 48 h incubation with SERMs, SERDs and selective AEBS/ChEH ligands were determined and quantified. Analyses were performed by HPLC and GC/MS as described in the Methods section. The cholesterol intermediates were quantified as a percent by the weight of total sterol. The amount of zymostenol and desmosterol was quantified by references to an external standard. C) HMCLs were treated with EtOH, OHT (10 μM), Tam (10 μM), RU 58668 (10 μM) or PBPE (40 μM) for 24 h and incubated with 0.6 μM [^14^C]-EC for 48 h and ChEH activity was assayed by measuring the conversion of CE into CT by TLC. A representative autoradiogram from three independent experiments is shown. D) RPMI 8226 and LP-1 cells were pre-incubated with 500 μM Vit E, 1 mM NAC, 50 nM BAF A1 or 10 mM 3-MA and then challenged with OHT (10 μM) or PBPE (40 μM) for 72 h. Cell death was determined by Trypan blue exclusion. Data were expressed as the percentage of cell death relative to control cells that received OHT (10 μM) or PBPE (40 μM). Experiments were repeated three times in duplicate with comparable results. The data present ed here are the means ± SEM of all experiments. *, *p*<0.001.

The presence of MLBs and UVs in OHT-treated cells could reflect an increasing mass of free sterols and lipids in OHT-treated cells. Detection of free sterols in HMCLs was achieved using filipin labeling. As shown Figure 3A, perinuclear vesicles were stained by filipin in OHT- but not in EtOH- or RU 58668-treated cells. As previously described for BC cells [12], we confirmed that Tam, OHT, benzylphenoxy-ethyl-pyrrolidin (PBPE) and RU 39411 bind to the AEBS with high affinity in HMCLs while RU 58668 had no measurable affinity (Figure 3B). Free sterols accumulated also in HMCLs treated with OHT and AEBS ligands (Supplementary Figure 2). Tam and PBPE induced the accumulation zymostenol, OHT and RU 39411 induced the accumulation of desmosterol and, as expected, RU 58668 did not induce free sterol accumulation (Figure 3B). Moreover, OHT and PBPE induced the accumulation of sphingomyelin, which was correlative with the increase of cellular free sterols (Supplementary Figure 2). Altogether, these data established that MLBs content found in HMCLs is similar to that found in BC cells [14]. Neutral lipids analyses showed that OHT and PBPE treatments induced the accumulation of triacylglycerol (TG) in cells explaining the presence of UVs (Supplementary Figure 2). We recently reported that TG biosynthesis and accumulation was mainly due to the inhibition of ChEH carried out by the AEBS, which led also to the accumulation of 5,6-ECs [18]. We next evaluated the activity of the ChEH enzyme and firstly verified that ChEH activity was measurable in each cell line. As shown Figure 3C, 5,6-EC accumulated in OHT-, PBPE- and Tam-treated cells indicating that ChEH activity was inhibited by these drugs. By contrast, its activation product, CT accumulated in EtOH- and RU 58668-treated cells (Figure 3C). We found that, in HMCLs, AEBS ligands inhibited ChEH in a dose-dependent fashion with submicromolar concentrations of IC_50_ except for RU 58668 which did not inhibit ChEH up to 10 μM (Table 1).

**Table 1:** Effect of drugs on 5,6-EC biosynthesis and unilamellar vesicles, multilamellar bodies, autophagic vesicles formation

Cell line	Drugs	ChEH IC50 (nM)	Vit E	MLB	AV	UV	5,6-EC
RPMI 8226	OHT	432 ± 14	− +	+ +	+ +	+ −	+ −
	Tam	52 ± 9	− +	+ +	+ +	+ −	+ −
	PBPE	927 ± 24	− +	+ +	+ +	+ −	+ −
	RU 39411	632 ± 22	− +	+ +	+ +	+ −	+ −
	RU 58668	>10,000	− +	− −	+ +	− −	− −
LP-1	OHT	551 ± 25	− +	+ +	+ +	+ −	+ −
	Tam	64 ± 11	− +	+ +	+ +	+ −	+ −
	PBPE	884 ± 32	− +	+ +	+ +	+ −	+ −
	RU 39411	727 ± 31	− +	+ +	+ +	+ −	+ −
	RU 58668	>10,000	− +	− −	+ +	− −	− −

The activity of ChEH was measured on intact cells. Cells were incubated with [^14^C]-5,6-EC (0.6 μM, 20 μCi/μmol) and were treated with increasing concentrations of drugs ranging from 1 nM to 10 μM over 24 h. IC_50_ represents the concentration of drugs required to inhibit 50% of ChEH activity. Cells were treated with 10 μM Tam, 10 μM OHT, 40 μM PBPE, 10 μM RU 39411 or 10 μM RU 58668 in the presence (+) or in the absence (-) of 500 μM Vit E for 48 h. Cells were analyzed by electron microscopy for the presence of multilamellar bodies (MLBs), autophagic vesicles (AVs), and unilamellar vesicles (UVs). About 100 cells per grid were observed for each condition. The sign + means that 80 % cells were positive and contained at least 5 vesicles otherwise they were considered as negative (-). The presence of 5,6-EC in cells was determined by GC/MS as described in the Methods section.

### AEBS ligands induce a protective autophagy in HCMLs

**Figure 4:**
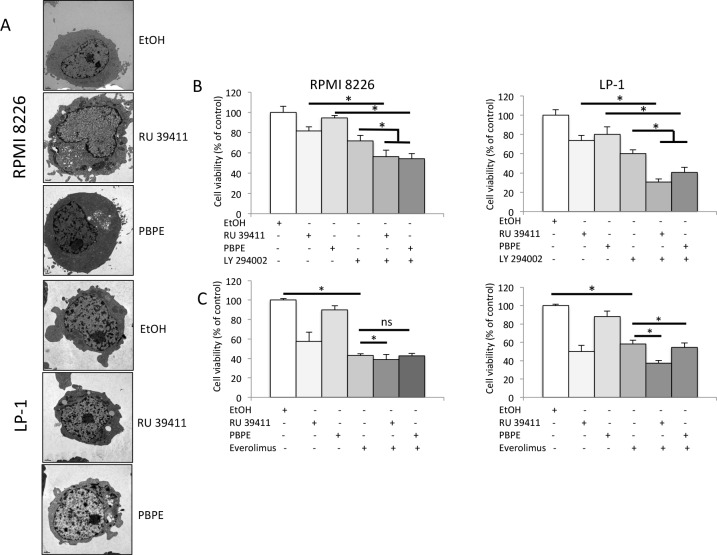
AEBS ligands induce PI3K/AKT/mTOR-mediated autophagy in HMCLs A) HMCLs were treated with RU 39411 (10 μM) or PBPE (40 μM) for 24 h and analyzed by transmission electron microscopy. B) and C) RPMI 8226 and LP-1 cells were cultured with (or without) 1 μM LY 294002 for 2 h (B), or 10 nM everolimus (RAD 001) for 2 h (C) then treated (or not) with 10 μM RU 39411 or 40 μM PBPE for 24 h (B) or 48 h (C). The number of viable cells was then determined with a MTT assay. Each experimental condition was repeated five times; the experiment was repeated three times. Plotted values (mean ± SD) are the percentage of viable cells referring to control experiments assigned to 100%. * *p*<0.05; ns, not significant.

As OHT, PBPE stimulated the rate of long-lived protein degradation in both cell lines. This stimulation was inhibited by a co-treatment with 3-methyladenosine (3-MA), a well-known inhibitor of autophagy (Figure 2D). Moreover, we observed the characteristics of autophagy in RU 39411- or PBPE-treated cells (Figure 4A and Supplementary Figure 3A). Although more subtle than for OHT, RU 39411 and PBPE treatments caused the formation of UVs, MLBs and autophagosomes (Table 1). The co-treatment of HCMLs with vitamin E (Vit E), a lipophilic antioxidant inhibited the formation of UV further confirming the role of sterol oxidation in the triggering of autophagy (Table 1). We previously observed in BC cells, that Vit E inhibited the specific stimulation of 5,6-EC production and TG biosynthesis by AEBS ligands while it had no impact on autophagosomes production or MLBs accumulation [12, 18]. We reported here that the co-treatment of HMCLs with AEBS ligands and Vit E induced a similar effect in RPMI 8226 and LP-1 cells strongly suggesting a similar mechanism (Table 1). Moreover, Vit E blocked the induction of cell death by AEBS ligands while NAC another antioxidant did not protect cells (Figure 3D). Since the inhibitors of autophagy (BAF A1 and 3-MA) increased the cytotoxicity of drugs, autophagy seemed a survival process in HMCLs. As a whole, AEBS ligands belonging to various structural classes (diphenyl-methane compounds, SERMs) induce similar effects on cholesterol metabolism and autophagy in HMCLs.

### The PI3K/AKT/mTOR pathway controls AEBS-induced autophagy

We next focused on the effects of the highly specific AEBS ligands, RU 39411 and PBPE and verified that they were capable of inducing a cell death that was strictly caspase-independent (Supplementary Figure 3B). The PI3K/AKT/mTOR axis is the master negative regulator of autophagy in various cell systems including MM, although constitutively activated in those cells [19-22].To ensure that the PI3K/AKT/mTOR pathway was recruited after AEBS ligand treatment, we used specific PI3K and mTOR inhibitors in combination with RU 39411 and PBPE. As shown Figure 4B, and in agreement with the known effects of PI3K inhibitors as inducers of apoptosis in HMCLs [23], LY 294002 induced cell death. Importantly, LY 294002 co-operated with RU 39411 and PBPE to enhance cell death. We concluded that LY 294002 triggered apoptosis and simultaneously, RU 39411 and PBPE induced autophagy through parallel pathways in both cell lines. The effect of everolimus was obviously different. Such as rapamycin another inhibitor of mTOR, everolimus induced cell death (Figure 4C). However, there was no clear additive effect of everolimus (even statistically significant for some combinations) when associated with AEBS ligands (Figure 4C). Our data suggest that everolimus and RU 39411/PBPE used similar or overlapping pathways to conduct to cell death/survival.

### Treatment of MM cells with AEBS ligands bypasses resistance to dexamethasone

**Figure 5:**
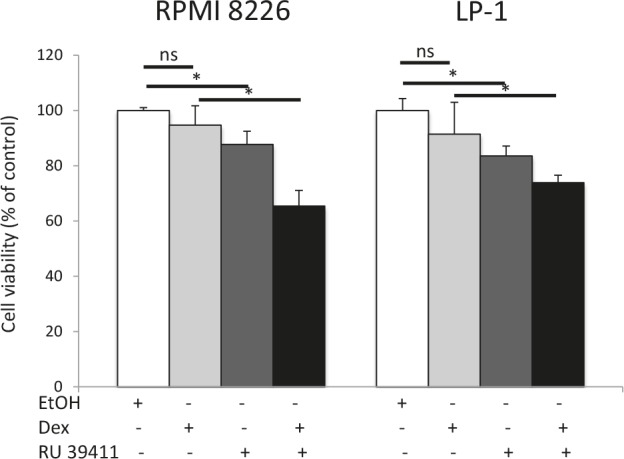
AEBS ligand alleviates dexamethasone resistance RPMI 8226 and LP-1 cells were cultured with vehicle (EtOH), RU 39411 (10 μM for 2 h or not) and then with 10 μM dexamethasone (Dex) for 24 h. The number of viable cells was then determined with a MTT assay. Each experimental condition was repeated three times. Plotted values (mean ± SD) are the percentage of viable cells referring to control experiments assigned to 100%. * *p*<0.05; ns, not significant.

Dexamethasone is widely used in clinical protocols for MM [2]. Interestingly, RPMI 8226 and LP-1 cells are both resistant to dexamethasone. To determine if the triggering of autophagy could restore sensitivity, RU 39411 was used to prime autophagy then dexamethasone was added to cultured HCMLs. The response was then evaluated by MTT assay as before. As shown Figure 5, the triggering of autophagy by RU 39411 restores the sensitivity of both cell lines to dexamethasone.

## DISCUSSION

Tamoxifen is the prototypical AE clinically used since more than 30 years for endocrine therapy and chemoprevention of ERα-positive breast cancers [24, 25]. Like its active metabolite OHT, Tam and a plethora of other SERMs bind to ER and inhibit tumor growth *via* gene transcription and non-genomic ER activities. They also affect other intracellular targets such as the AEBS/ChEH [26]. SERMs inhibit the growth of HMCLs and induce their apoptosis through the intrinsic mitochondrial pathway [5]. In the present work, we provide evidences for the induction of a sterol-dependent protective autophagy by several AEBS ligands. Similarly to what we reported for ERα-positive BC cells, we found that this activity proceeds through alteration of cholesterol metabolism [14].

MM cells synthesize large amounts of immunoglobulins and activate the unfolded protein response (UPR) pathway. Autophagy is a protective process by which malignant MM cells protect themselves from unfolded or misfolded proteins[15] and compromising the UPR cascade induces an autophagic cell death in MM cells [27]. Moreover, targeting autophagy in MM sensitizes cells to various drugs such as new proteasome inhibitors [28], nucleoside analogue [29], HSP90 inhibitors [20], and DNA-damaging agents [30]. Targeting autophagy has been proposed for fighting myeloma [31]. As a whole, constitutive autophagy is a survival mechanism for MM cells although excessive autophagy, induced by several stresses including drugs, is a mechanism of cell death. We show here that AEBS ligands such as OHT, Tam, RU 39411 and PBPE are able to induce a protective autophagy in MM cells through a mechanism similar to what was found in BC cells. We observed that AEBS ligands, including Tam and OHT, induced a caspase-independent cell death through a mechanism that required the production and the accumulation of 5,6-EC [14, 18, 32]. Interestingly, we observed the same response in the two HMCLs we used, despite their opposite *RAS* status (mutated for RPMI 8226 and wild-type for LP-1, [33]) and their belonging to different prognostic groups according to the UAMS classification [34]. Importantly, the activation of autophagy alleviates resistance to dexamethasone. Our data support the notion of autophagy as a survival mechanism in MM cells. Moreover, AEBS ligands alone or in combination with dexamethasone could represent a useful anti-myeloma therapy.

Metabolic alterations are hallmarks of cancer including MM, and participate in the resistance towards apoptosis inducers. It has been shown that targeting glucose metabolism induces autophagy and markedly enhances cell death in HMCLs [29]. Our study establishes that altering cholesterol metabolism is a mean to induce cell death and to restore sensitivity in HMCLs. Indeed, AEBS ligands inhibit post-lanosterol cholesterol enzymes, further conducting cells to accumulate cholesterol precursors and sphingomyelin leading to MLB formation and to autophagy. We have described the same features for BC cells [18]. Nonetheless, in BC cells, Tam induces cell death through a mechanism of differentiation driven by the generation of reactive oxygen species (ROS) and 5,6-EC production. Although, the inhibition of lipid oxidation enhanced AEBS-mediated cell death in HMCLs, no differentiation process could occur, HMCLs being at the last step of differentiation [35]. In BC cells, accumulation of 5,6-ECs (5,6α-EC and 5,6β-EC) contributes to cytotoxic, antiproliferative and chemopreventive effects of ChEH inhibitors [36]. Moreover, 5,6 α-EC is an endogenous ligand for the liver X receptors (LXR) α and β [37] and LXRs participate in cholesterol transport and metabolism as well as the regulation of tumor cell growth [38]. Although not analyzed in the present study, LXRβ are known to be overexpressed in MM cells [39]. Moreover, in good agreement with previous study [18], TG was produced in response to the accumulation of 5,6-EC after AEBS ligand treatment. We can therefore hypothesize that LXRβ are stimulated by the AEBS in HMCLs. In another hand, we found that 5,6β-EC was responsible of a mitochondrial cytotoxicity in BC cells and that overexpression of BCL2 protected cells against the cytotoxicity induced by 5,6β-EC as well as SERMs and selective AEBS ligands [18]. Although, the antiapoptotic protein BCL2 is overexpressed in MM, RPMI 8226 and LP-1 cells respond to AEBS ligands.

MM cells are particularly armed to counteract various stresses. In turn, the inhibition of proteasome degradation, aggresome formation, heat shock proteins accumulation or UPR pathway leads to autophagy [15, 19-22]. It is tempting to speculate that the accumulation of UVs, MLBs and free cholesterol in MM cells could be sufficient to induce an autophagic response and that the level of this response governs survival or death. Alteration of cholesterol trafficking, accumulation of free sterol and formation of MLBs lead to autophagy-mediated neurodegeneration in the Niemann-Pick type C disease [40, 41]. Lipophagy has been described as an alternative pathway of lipid metabolism [42]. In this form of metabolism, cholesterol is taken up by autophagosomes and delivered to lysosome degradation. Impaired lipophagy could sensitize MM cells to death stimuli.

Besides the well-known role of mTOR in the inhibition of autophagy, an emerging role is the control of lipid metabolism and storage [43, 44]. However, the signaling routes for both effects are still poorly defined. Our data could suggest that the control by mTOR of both autophagy and cholesterol metabolism are affected by AEBS ligands in HMCLs. Further efforts are needed to characterize their downstream effectors within the mTOR pathway(s). This may conduct to discover new targets potentially involved in the treatment of myeloma but also in other malignancies in which lipid metabolism is deregulated such as diabetes and obesity.

## METHODS

### Chemicals

4-[(Z)-1-[4-(2-dimethylaminoethyloxy)phenyl]-2-phenyl]-1-but-1-enyl]-phenol or OHT and [quinoyl-valyl-O-methylaspartyl-(2,6-difluoro-phenoxy)-methyl ketone] (Q-VD-OPh), BAF A1, MDC, filipin, everolimus (or RAD-001), LY 294002, dexamethasone, Vit E or α-tocopherol, NAC, 3-MA and oxysterols were purchased from Sigma-Aldrich (Saint-Quentin Fallavier, France). [^14^C]-labelled 5,6-EC and CT were synthesized as reported before [18]. 17β-11-[4-[5-[(4,4,5,5,5-pentafluoropentyl)-sulfonyl]-pentyloxy]-phenyl]estra-1,3,5(10)-triene,3,17-diol or RU 58668 and 11β-(4-(2-(dimethylamino)-ethoxy)-phenyl)estra-1,3,5(10)-triene-3,17β-diol or RU 39441 were gifts of P Van de Velde (Sanofi, Paris, France). Fulvestrant (ICI 182,780) was obtained from AstraZeneca (Macclesfield, UK). 1-(2-(4-benzylphenoxy)-ethyl)-pyrrolidin-HCl (PBPE) was synthesized as described [45]. Chemical structures of drugs are presented in Supplementary Figure 4.

### Cell cultures, cell treatments and viability measurement

RPMI 8226 were obtained from R Bataille (CLCC, Nantes, France) and LP-1 cells from D Bouscary (Institut Cochin, Paris, France). At their arrival, the identity of cells was checked by karyotyping and surface markers expression; cells were then amplified and frozen. HMCLs were maintained in RPMI 1640 medium (Lonza, Basel, Switzerland) supplemented with 2 mM L-glutamine (Lonza) and 10% FCS (fetal calf serum) (PAA Laboratories, Velizy-Villacoublay, France). In experiments with AEs, cells were cultured in phenol red-free medium plus FCS. AEs were dissolved in ethanol (EtOH) to obtain 10 mM stock solution and then diluted into medium immediately before use. For controls, vehicle was added at the same final concentration. Cell viability was determined using CellTiter 96® AQueous One Solution (MTT [(3-(4,5)-dimethylthiazol-2-yl)-2,5-diphenyltetrazolium bromide] assay, Promega, Charbonnières, France) according to the supplier's instructions.

### Confocal fluorescence microscopy analysis

Cells were cytospinned on superfrost glass slides at 500 g for 3 min, then fixed in 4% paraformaldehyde (PFA) and permeabilized with 0.5% Triton-X100 (v/v) for 5 min. Slides were incubated with primary anti-MAP LC3 (H-50, Santa Cruz Biotechnologies, Heidelberg, Germany) antibody (Ab) and as fluorescent secondary Ab (AbII) Dylight™488 anti-rabbit IgG (Rockland) as previously described [14]. Incubation with AbII alone served as negative control. Slides were mounted and analyzed with the Fluoview FV 1000 confocal microscope and Fluoview Viewer software (Olympus, Rungis, France). The detection of autophagic vacuoles was done by MDC staining as previously described [14]. Filipin staining was done by incubating glass slides for 1 h at room temperature with 50 μg/ml filipin in PBS solution as described [14]. Staining was visualized using a Nikon eclipse 90i.

### Apoptosis determination by APO2.7 staining and cytometry sorting

HMCLs exposed to vehicle (ethanol, EtOH) or OHT (10 μM for 24-72 h) were stained with Apo 2.7 PE-conjugated Ab (Beckman Coulter, Villepinte, France). APO2.7-stained HMCLs were analyzed by flow cytometry (Gallios, Beckman Coulter) and data were processed with the Kaluza software (Beckman Coulter). On average, 2 × 10^4^ cells were analyzed for each culture condition, for each experiment.

### Measurement of mitochondrial membrane potential

Treated HMCLs were stained with 3,3'-dihexyloxacarbocyanide iodide (DiOC_6_(3), Molecular Probes®) which incorporates into internal mitochondria membrane of healthy cells. The reduction of ΔΨm representative of apoptosis induction was quantified by the decrease of DiOC_6_(3) retention as described [5]. On average, 2 × 10^4^ cells for each sample, for each condition, were acquired and sorted by flow cytometry (Gallios, Beckman Coulter) and data were processed with the Kaluza software (Beckman Coulter).

### Transmission electronic microscopy analysis

HMCLs were fixed with 2.5% glutaraldehyde in 0.1 M Sorensen phosphate buffer pH 7.4 and post-fixed with 1% OsO_4_ in the same buffer. Cells were then dehydrated through a graded ethanol series, embedded in Epon 812 and cut into thin sections (80 nm). Sections were stained with uranyl acetate and lead citrate, observed with a JEOL 1011 transmission electron microscope equipped with a Megaview III camera.

### Western blotting

Whole cell lysates and western blotting were prepared as previously described [5]. We used primary Ab against LC3 (H-50, Santa Cruz Biotechnologies) and ImmunoPure goat anti-rabbit IgG peroxidase-conjugated as AbII (Pierce Protein Research Products, Thermo Fisher Scientific, Rockford, IL).

### Analysis of protein degradation

The degradation of long-lived proteins was measured as described previously [46]. Briefly, HMCLs were incubated for 24 h at 37°C with 0.2 μCi/ml of L-[^14^C]valine in complete medium. Cells were then washed resuspended in medium with no nutrients but supplemented with 10 mM valine. After the first h of incubation, the chase medium was replaced by complete medium and the incubation continued for 5 h. When required drugs (10 μM OHT, 40 μM PBPE or 10 mM 3-MA) were added to the chase medium. Radiolabeled proteins present in the chase medium as well as in cells were precipitated with 10% trichloroacetic acid (v/v) at 4°C. Radioactivity was measured by scintillation counting. The rate of protein degradation was calculated from the ratio of radioactivity in the medium to that of precipitated cell fraction.

### Lipid analysis

HMCLs (10^8^ cells) were treated with the solvent vehicle, 10 μM PBPE or 2.5 μM OHT for 48 h then washed and lipids were extracted with chloroform/methanol (2/1). The organic layer was reduced to dryness under a flux of argon and the residue was resuspended in ethanol. Sterols were analyzed by liquid chromatography and gas-liquid chromatography as described before [12]. Neutral lipids and sphingomyelin analyses were done according to previously published procedures [14, 18, 47].

### ChEH activity assay

HMCLs cells were seeded in 6-well plates (0.5 × 10^6^ cells/well) in complete medium and incubated with 0.6 μM [^14^C]-5,6α-EC in the presence of EtOH, OHT (10 μM), Tam (10 μM), RU 58668 (10 μM) or PBPE (40 μM) for 24 h. Cells were then pelleted and extracted with chloroform/methanol (2/1). The organic layer was reduced to dryness under a flux of argon and the residue was resuspended in EtOH. Samples were applied to thin layer chromatography (TLC) plates and developed using ethyl acetate as previously described [14, 18]. The radioactive metabolites were visualized by autoradiography using Kodak Biomax MS films.

### Statistical analysis

Student's *t*-test was used to determine the significance of differences between two experimental groups. Data were analyzed with a two-sided test and *p*<0.05 was considered significant.

## SUPPLEMENTARY FIGURES AND TABLES




