# Supplementary material for: Antiestrogen-binding site ligands induce autophagy in myeloma cells that proceeds through alteration of cholesterol metabolism
Source: Oncotarget. 2013 Jun 11;4(6):911–22. doi: 10.18632/oncotarget.1066 (PMC3757248; doi:10.18632/oncotarget.1066)
Supplement: Supplementary file 1 [file oncotarget-04-911-s001.pdf]

Antiestrogen-binding site ligands induce autophagy in myeloma cells that proceeds through alteration of cholesterol metabolism - Sola et al

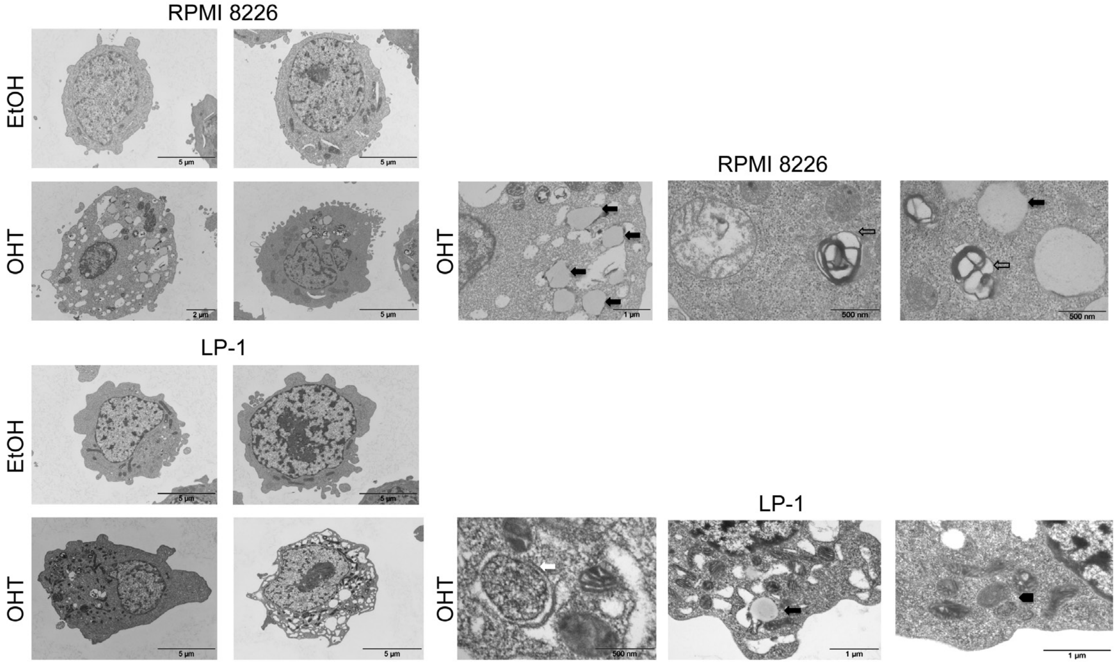

Figure S1: OHT-treatment induces autophagy in MM cells. RPMI 8226 and LP-1 cells were treated with EtOH or OHT (10 µM) for 24 h and analyzed by transmission electron microscopy. The visualization at high magnification of the cytosolic vesicles allowed the identification of unilamellar vesicles (UV, black arrows), multilamellar bodies (MLB, open arrows), autophagosomes (white arrows) and autolysosomes (arrowhead).

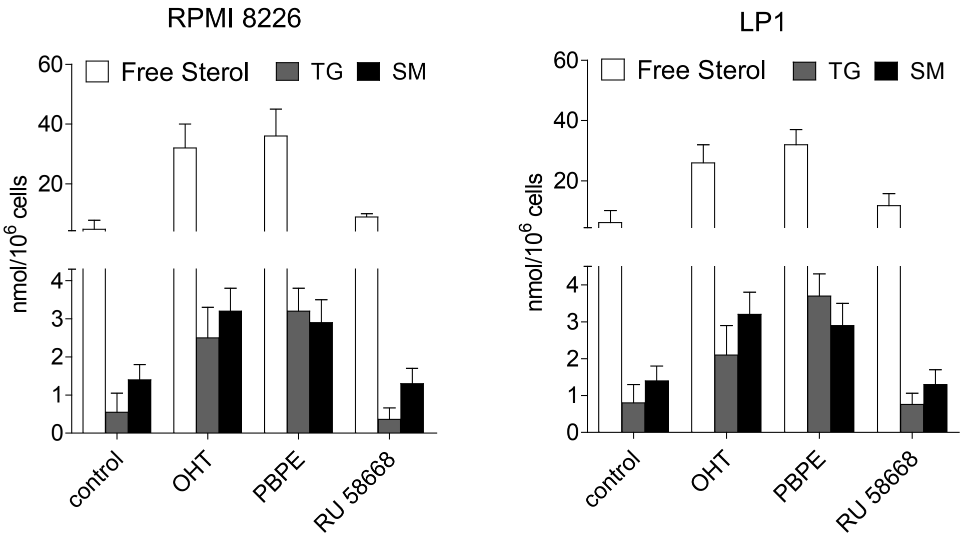

Figure S2: Treatment of MM cells by OHT and PBPE but not RU 58668 increases intracellular free sterol, triacyl glycerol(TG) and sphingomyelin(SM) contents. RPM 8226 and LP-1 cells (108, 8) were treated with solvent vehicle (control), 5 µM OHT, 20 µM PBPE or 5 µM RU 58668 for three days. Sterol and lipid content was carried out as described in Methods section. Mean ± SD of three independent experiments.

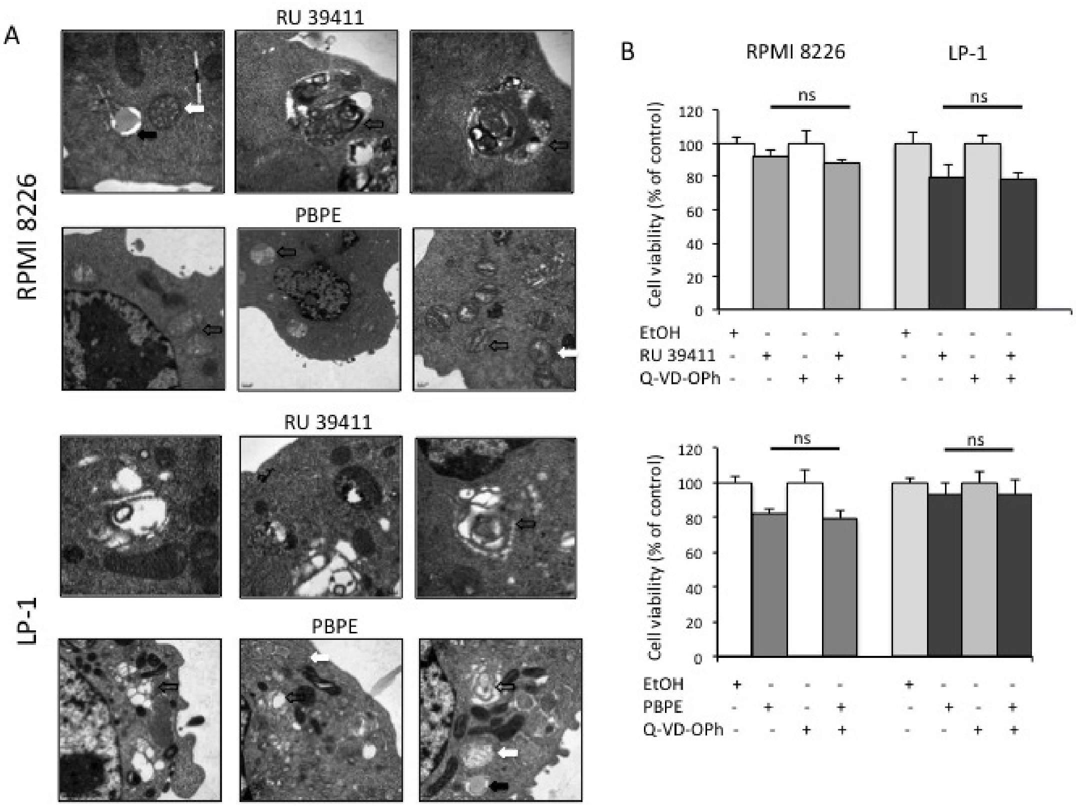

Figure S3: AEBS ligands induce caspase-independent cell death and autophagy in MM cells. A) RPMI 8226 and LP-1 cells were treated with RU 39411 (10 µM) or PBPE (40 µM) for 24 h and analyzed by transmission electron microscopy. The visualization at high magnification of the cytosolic vesicles allowed the identification of unilamellar vesicles (black arrows), multilamellar bodies (open arrows) and autophagosomes (white arrows). B) RPMI 8226 and LP-1 cells were cultured with (or without) 10 µM Q-VD-OPh for 2 h, then treated (or not) with 10 µM RU 39411 (upper part) or 40 µM PBPE (lower part) for 48 h. The number of viable cells was then determined with a MTT assay. Each experimental condition was repeated five times; the experiment was repeated twice. Plotted values (mean ± SD) are the percentage of viable cells referring to control experiments assigned to 100%. ns, not significant with the Student's t-test.

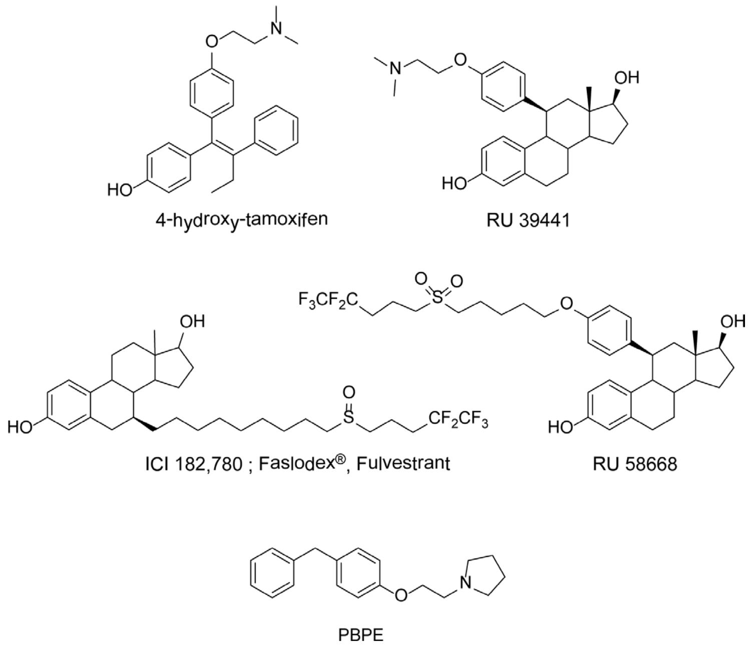

Figure S4: Chemical structures of drugs used in the study. 4-hydroxytamoxifen, RU 39441 and PBPE are all high affinity AEBS ligands and ChEH inhibitors; RU 58668 and ICI 182,780 (or fulvestrant) are not. For further informations on the drugs see e.g (13 and Poirot M et al. Curr Opin Pharmacol 2012;12:683-9).
