# Supplementary material for: Antiestrogen-binding site ligands induce autophagy in myeloma cells that proceeds through alteration of cholesterol metabolism
Source: Oncotarget. 2013 Jun 11;4(6):911–22. doi: 10.18632/oncotarget.1066 (PMC3757248; doi:10.18632/oncotarget.1066)
Supplement: Supplementary file 2 [file oncotarget-04-911-s002.docx]

**Antiestrogen-binding site ligands induce autophagy in myeloma cells that proceeds through alteration of cholesterol metabolism - Sola et al**

***Supplementary Table***

**Table S1. SERM and SERDs effects on MM cell lines**

| **Cell lines** | **OHT** | **RU 58668** | **Fulvestrant** |
| --- | --- | --- | --- |
| Karpas 620 | No response | No response | Not done |
| *LP-1* | *G1-arrest and apoptosis* | G1-arrest | G1-arrest |
| NCI-H929 | G1-arrest and apoptosis | Apoptosis | Apoptosis |
| OPM-2 | No response | G1-arrest | G1-arrest |
| *RPMI 8226* | *G1-arrest and apoptosis* | Apoptosis | Apoptosis |
| U266 | G1-arrest and apoptosis | No response | Not done |

In italic, cell lines that have been used in the present study.
